# Fluorescent In Situ Staining and Flow Cytometric Procedures as New Pre-Diagnostic Tests for Sialidosis, GM1 Gangliosidosis and Niemann–Pick Type C

**DOI:** 10.3390/biomedicines10081962

**Published:** 2022-08-12

**Authors:** Claudia Capitini, Federica Feo, Anna Caciotti, Rodolfo Tonin, Matteo Lulli, Domenico Coviello, Renzo Guerrini, Martino Calamai, Amelia Morrone

**Affiliations:** 1 European Laboratory for Non-Linear Spectroscopy (LENS), University of Florence, 50019 Sesto-Fiorention, Italy; 2 National Institute of Optics-National Research Council (CNR-INO), 50019 Sesto Fiorentino, Italy; 3 Laboratory of Molecular Biology of Neurometabolic Diseases, Neuroscience Department, Meyer Children’s Hospital, Viale Pieraccini n. 24, 50139 Florence, Italy; 4 Department of Experimental and Clinical Biomedical Sciences “Mario Serio”, University of Florence, 50134 Florence, Italy; 5 Laboratory of Human Genetics, IRCCS Istituto Giannina Gaslini, 16147 Genoa, Italy; 6 Department of Neurosciences, Psychology, Pharmacology and Child Health, University of Florence, 50121 Florence, Italy

**Keywords:** GM1 gangliosidosis, sialidosis, Niemann–Pick type C, lysosomal storage disorders, flow cytometry, fluorescent imaging, Cholera Toxin B, Filipin, cholesterol, sialic acid, biomarkers

## Abstract

Background: Early diagnosis is essential in the field of lysosomal storage disorders for the proper management of patients and for starting therapies before irreversible damage occurs, particularly in neurodegenerative conditions. Currently, specific biomarkers for the diagnosis of lysosomal storage disorders are lacking in routine laboratory practice, except for enzymatic tests, which are available only in specialized metabolic centers. Recently, we established a method for measuring and verifying changes in GM1 ganglioside levels in peripheral blood lymphocytes in patients with GM1 gangliosidosis. However, fresh blood is not always available, and using frozen/thawed lymphocytes can lead to inaccurate results. Methods: We used frozen/thawed fibroblasts obtained from stored biopsies to explore the feasibility of fluorescent imaging and flow-cytometric methods to track changes in storage materials in fibroblasts from patients with three lysosomal neurodegenerative conditions: GM1 gangliosidosis, Sialidosis, and Niemann–Pick type C. We used specific markers for each pathology. Results and Conclusions: We demonstrated that with our methods, it is possible to clearly distinguish the levels of accumulated metabolites in fibroblasts from affected and unaffected patients for all the three pathologies considered. Our methods proved to be rapid, sensitive, unbiased, and potentially applicable to other LSDs.

## 1. Introduction

Glycoproteinosis sialidosis and sphingolipidosis GM1 gangliosidosis are closely related both clinically and biochemically. The causal deficient enzymes, neuraminidase (NEU1) and beta-galactosidase (GLB1) interact in a multienzyme complex. NEU1 and GLB1 gain a stable conformation by virtue of their association with the protective protein cathepsin A (PPCA) in the lysosomal complex. The pleiotropic NEU1 enzyme expresses all of its catalytic activity in the NEU1-PPCA-GLB1 complex, i.e., the cleavage of terminal N-acetylated neuraminic acids (sialic acid), glycoproteins, glycolipids, and oligo- and polysaccharides [1]. PPCA stabilizes the lysosomal complex and ensures the correct function of GLB1 that can remove the terminal β-1,4-linked galactose residue from glycoproteins, glycosaminoglycans, and above all, gangliosides, specifically GM1 ganglioside. Unesterified cholesterol and sphingolipids, including GM1 ganglioside, accumulate in the sphingolipidosis Niemann–Pick type C (NPC) [2,3].

These three neurodegenerative conditions are very rare, with an approximate prevalence of 1/5,000,000-1/1,500,000 live births for Sialidosis (OMIM #256550)[4], 1:100,000–1:200,000 live births for GM1 gangliosidosis (OMIM #230500) [5,6], and from 0.35 to 2.2 per 100,000 births, depending on the geographic area, for NPC (OMIM #257220) [7]. The mild form of sialidosis Type I, whose clinical presentation is mostly confined to ophthalmologic problems and myoclonus, and the juvenile/adult forms of GM1 gangliosidosis are probably underestimated and likely to be more common than reported [8,9].

The clinical course of GM1 gangliosidosis always involves progressive neurodegeneration in the more severe infantile form, in the late infantile/juvenile form, and in patients with adult-onset, in whom extrapyramidal signs prevail. The cherry red spot, facial dysmorphism, visceromegaly, and cardiomyopathy are much more frequent in the severe forms [10].

Sialidosis has two main types. The dysmorphic type II form is characterized by congenital hydrops, hepato-splenomegaly, and severe neurological involvement, while the normosomatic type I form is mainly characterized by visual defects, ataxia, and myoclonus [11]. Three phenotypes of NPC can be distinguished based on the age of neurological onset, which can occur in early infancy, childhood, or adolescence/adulthood, and the rate of neurodegenerative progression, which ranges from a devastating onset to slow progression [12].

The biochemical characterization of NPC patients has traditionally been performed by the evaluation of cholesterol accumulation via the “Filipin test”, which, however, is not resolutive in up to 15% of patients [2]. For the diagnosis of GM1 gangliosidosis and sialidosis, a biochemical evaluation of urinary oligosaccharides and enzyme assays using fluorogenic (4-methylumbellyferyl-) substrate is routinely performed in specialized metabolic centers. More recently, the use of capillary electrophoresis with laser-induced detection and mass spectrometry (LC-ESI-MS) techniques have been adopted [13]. Glycosphingolipid (GSL) evaluations can be used in the diagnosis of sphingolipidoses; however, most current techniques, including LC-ESI-MS, require extensive sample preparation and/or expensive MS/MS facilities [14].

Several biomarkers have been investigated to assess their ability to diagnose GM1 gangliosidosis and NPC, track disease progression and monitor therapeutic strategies [9,12,15]. In particular, cholestane-3β,5α,6β-triol (C-triol), trihydroxycholanic acid glycinate (TCG), and N-palmitoyl-O-phosphocholineserine (PPCS, initially referred to as lysoSM-509) have led to the development of blood-based diagnostics for NPC [16]. Substrate reduction therapy using Miglustat [N-Butyl-Deoxynojirimycin (NB-DNJ)], as a central nervous system modifier, has been approved for NPC in several countries [6] and has also been used in GM1 gangliosidosis [17]. However, current therapeutic strategies for NPC, sialidosis, and GM1 gangliosidosis remain unsatisfactory.

We propose fluorescent imaging and flow-cytometric methods to track changes in GM1 ganglioside, sialic acid, and cholesterol levels in patients with the above-mentioned LSDs. Alternative diagnostic tools for these pathologies provide a significant step forward for their correct identification and management.

## 2. Materials and Methods

### 2.1. Patients, Ethical Statements, and Cell Cultures

All of the patients were diagnosed at a clinical, biochemical, and molecular level. Their details are presented in Table 1. The patients had been previously described (Pt1 in [18]; Pt2 in [19], Pt 3 in [20], Pt4 in [11], and Pt 5 in [21]. NPC cells were obtained from the “Cell Line and DNA Biobank from Patients Affected by Genetic Diseases” (G. Gaslini Institute)—Telethon Genetic Biobank Network [22].

The isolated skin fibroblasts from patients were cultured in Dulbecco’s modified Eagle’s medium with 10% fetal bovine serum and 1% penicillin/streptomycin antibiotics solution.

### 2.2. GM1 Gangliosidosis Cell Imaging

Fibroblasts from GM1 gangliosidosis patients and from the wild-type (WT) control were plated in 12-well plates containing glass coverslips at a 30,000 cells/well density. Twenty-four hours after plating, the cells were fixed with 4% PFA, rinsed with PBS (plus 0.5 mM MgCl_2_ and 0.8 mM CaCl_2_), and permeabilized with 0.075% Triton X. After rinsing with PBS and blocking with 4% BSA-PBS, the cells were incubated for 30 min with 10 µg/mL Cholera Toxin B subunit and FITC conjugate (FITC-CTXb, Sigma-Aldrich Merck, Burlington, MA, USA) together with the Hoechst 33342 dye (10 µg/mL, Sigma-Aldrich Merck) to stain GM1 and the nuclei, respectively. The coverslips were then washed with PBS and water and mounted on a glass slide. Cell imaging was performed on a Nikon Eclipse TE300 C2 confocal laser scanning (CLSM) (Nikon, Tokyo, Japan) equipped with a Nikon 60x immersion oil objective (Apo Plan, NA 1.4) and with Coherent CUBE (diode 405 nm) and Melles Griot (Argon 488 nm) lasers. The emission filters for imaging were 452/45 nm and 514/30 nm. The settings were kept constant for each analysis. Fifty to sixty cells were analyzed for each examined sample by using ImageJ software (National Institutes of Health, Bethesda, MD, USA).

### 2.3. Sialidosis Cell Imaging

The fibroblasts from patients with sialidosis and from the WT controls were plated in 12-well plates containing glass coverslips at a 30,000 cells/well density. Twenty-four hours after plating, the cells were fixed, permeabilized, and blocked, as described above, and then they were incubated for 30 min with 5 µg/mL of Wheat Germ Agglutinin and Alexa Fluor™ 594 Conjugate (WGA-594, Thermofisher, Waltham, MA, USA) to stain the sialic acid and with the Hoechst 33342 dye. After rinsing with PBS and water, the coverslips were mounted on a glass slide and imaged with a CLSM equipped with Coherent CUBE (diode 405 nm) and Coherent Sapphire (Sapphire 561 nm) lasers. The emission filters for imaging were 452/45 nm and 595/60 nm. The settings were kept constant for each analysis. Fifty to sixty cells were analyzed for each examined sample by using ImageJ software.

### 2.4. NPC Cell Imaging

The fibroblasts from NPC patients and from the WT control were plated in 12-well plates containing glass coverslips at a 30,000 cells/well density. Twenty-four hours after plating, the cells were fixed, permeabilized, and blocked, as described above, and then they were separately incubated with 0.25 mg/mL of Filipin III (Sigma-Aldrich, St. Louis, MO, USA) for 24 h to stain the cholesterol and with FITC-CTXb and Hoechst 33342 for 30 min. After rinsing with PBS and water, the cells stained with Filipin III were imaged with a custom-made wide-field epifluorescence microscope equipped with an oil-immersion objective (Nikon Plan Apo TIRF 100x/1.45), a DC4100 4-Wavelengths LED Source (Thorlabs, Newton, NJ, USA) and a heating chamber. A 405 UV-LED with a BFP excitation and emission filter set (Chroma, Taoyuan, Taiwan) was used. Instead, the cells stained with FITC-CTXb and Hoechst 33342 were imaged with LSCM, as described above.

### 2.5. Flow Cytometric Analysis

The fibroblasts from patients with GM1 gangliosidosis, sialidosis, NPC, and the healthy WT controls were plated in 12-well plates at a 30,000 cells/well density. Twenty-four hours after plating, the cells were harvested and washed with PBS. A BD Cytofix/CytopermTM Fixation/Permeabilization Solution kit (BD Biosciences, Lake Franklin, NJ, USA) was used for fixation and permeabilization according to the manufacturer’s instructions. The fibroblasts from GM1 gangliosidosis were incubated with 10 µg/mL of FITC-CTXb prepared in a 1x BD Perm/Wash buffer containing FBS and saponin (provided along with the kit) for 30 min at room temperature. The fibroblasts from sialidosis were instead incubated with 5 µg/mL of WGA-594 in a 1x BD Perm/Wash buffer for 30 min at room temperature. Finally, the fibroblasts from the NPC patients were incubated separately with 0.25 mg/mL of Filipin III and 10 µg/mL of FITC-CTXb, in a 1x BD Perm/Wash buffer, for 24 h and 30 min, respectively, at room temperature. After washing with a 1x BD Perm/Wash buffer, the samples were analyzed on a FACSCanto II flow cytometer (BD Biosciences, Lake Franklin, NJ, USA). Data were analysed using the free Flowing software (Cell Imaging and Cytometry Core, Turku Bioscience Centre, Turku, Finland). The fibroblasts were identified by side-scattered (SSC) and forward-scattered (FSC) light. The GM1, sialic acid, and cholesterol content were quantified by the median fluorescence intensity (MFI) of the cellular population labeled with the corresponding markers. For each experiment, the WT control cells were labeled and analyzed under the same experimental conditions in parallel with the patient cells.

### 2.6. Statistics

The CSLM data were expressed as mean ± Standard Deviation (S.D.). Statistical significance was evaluated using a Student’s test for both the CLSM and flow cytometry data. A *p*-value lower than 0.05 was considered to be statistically significant. The single (*), double (**), and triple (***) asterisks refer to *p*-values lower than 0.05, 0.01, and 0.001. Statistical analysis was performed using the KaleidaGraph software.

## 3. Results

### 3.1. GM1 Gangliosidosis

**Figure 1:**
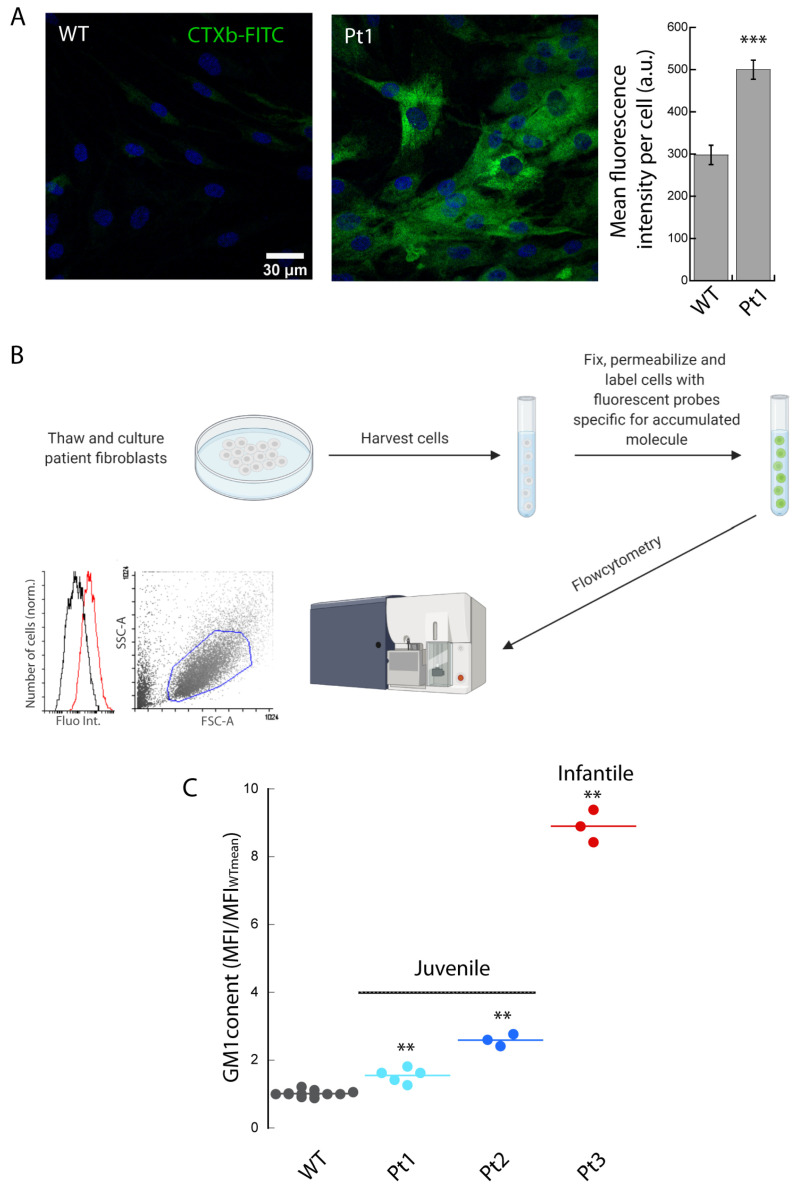
GM1 gangliosidosis. Thawed primary cultures of fibroblasts isolated from control and GM1 gangliosidosis patients were cultured, fixed, permeabilized, labeled with CTXb-FITC, and imaged with CLSM (**A**) or analyzed with flow cytometry (**B**,**C**). (**A**) The juvenile patient (Pt1) shows higher values of CTXb-FITC fluorescence intensity compared to WT, indicating an increase in GM1 content. Scale bar 30 µm. >20 cells were analyzed for each condition. Error bar S.D. (**B**) Schematic representation of the workflow to analyze the cells of affected and unaffected cells with flow cytometry. The FSC-A vs. SSC-A plot is gated (blue line) to exclude cellular debris, the corresponding CTXb-FITC fluorescence intensity distributions of affected (red line) and unaffected (black line) are analyzed, and median fluorescence values (MFI) are extrapolated. (**C**) MFI/MFI_WTmean_ values are obtained by dividing the MFI of a distribution by the mean MFI obtained from the controls. MFI/MFI_WTmean_ values increase proportionally to the severity of the pathology in juvenile (Pt1 and Pt2) and infantile patients (Pt3). >5000 cells were analyzed for each MFI. Student’s *t*-test ** *p* ≤ 0.01, *** *p* ≤ 0.001.

The fibroblasts from a juvenile patient (Pt1) and healthy control (WT) were thawed, cultured, fixed, permeabilized, and labeled with the beta-subunit of cholera toxin (CTXb, which specifically binds to GM1 ganglioside) directly coupled to FITC. In agreement with previous results [9], in which we used biotinylated CTXb and streptavidin Alexa488 fluorophore, CLSM imaging and analysis revealed a ~70% increase in the mean fluorescence intensity in the fibroblasts from the juvenile patient affected by GM1 gangliosidosis with respect to the fibroblasts from an unaffected donor (Figure 1A).

To investigate whether flow cytometry could be used to rapidly detect changes in the levels of GM1 not only in lymphocytes [9] but also in adherent cells, we detached, fixed, permeabilized, and labeled the patient and control fibroblasts with CTXb-FITC (Figure 1B). The cells were discriminated from debris by FSC and SSC scatter gating, and the median fluorescence intensity (MFI) values used to compare the data. As previously described [9], we quantified the changes in fluorescence intensity distribution between the controls and the patients by normalizing the MFI values of the patient and wild-type (WT) control samples to the mean MFI value from the controls (MFI/MFI_WTmean_) that simultaneously underwent the same procedure of fixation and labeling. The juvenile Pt1 displayed a ~60% increase in MFI/MFI_WTmean_ compared to the control. A ~2.5-fold higher amount of GM1 was found in the fibroblasts originating from another juvenile patient (Pt2), while a notable ~8.5-fold increase was found in the fibroblasts from an infantile patient (Pt3), further corroborating the link between the severity of the disease and the accumulation of GM1 in the lymphocytes [9] (Figure 1C).

### 3.2. Sialidosis

**Figure 2:**
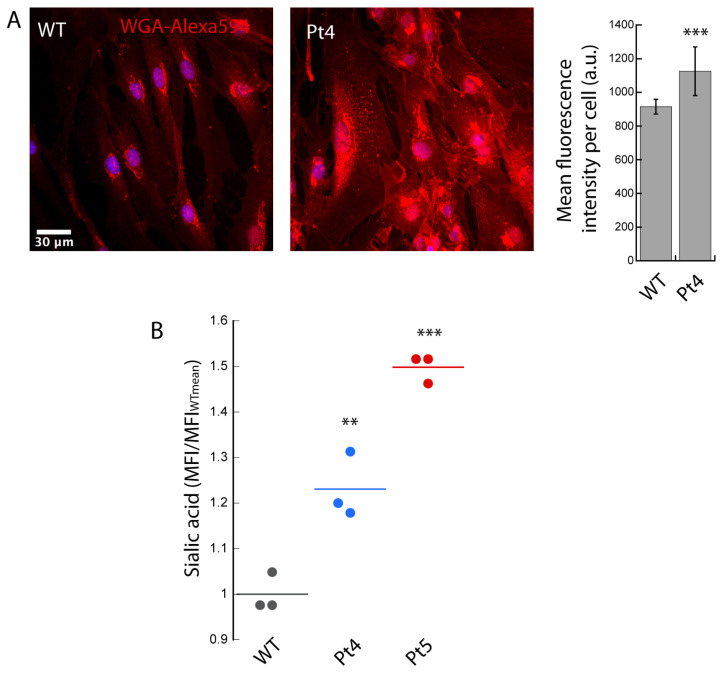
Sialidosis. Thawed primary cultures of fibroblasts isolated from control and Sialidosis patients (Pt4 and Pt5) were cultured, fixed, permeabilized, labeled with WGA-594, and imaged with CLSM (**A**) or analyzed with flow cytometry (**B**). (**A**) The Pt4 patient shows higher values of WGA-594 fluorescence intensity compared to WT control, indicating a rise in content of molecules containing sialic acid groups. Scale bar 30 µm. >20 cells were analyzed for each condition. Error bar S.D. (**B**) MFI/MFI_WTmean_ values increased in the fibroblast populations of both patients (Pt4 and Pt5) analyzed with respect to control. >5000 cells were analyzed for each MFI. Student’s *t*-test ** *p* ≤ 0.01, *** *p* ≤ 0.001.

We followed the same approach used on the fibroblasts from GM1 gangliosidosis patients for the fibroblasts derived from patients with Sialidosis. In this case, we used a fluorescently-labeled lectin, WGA, coupled to Alexa dyes, which specifically binds to N-acetyl-D-glucosamine and sialic acid. A preliminary test carried out with CLSM demonstrated that it is possible to detect differences between the WT control and patient fibroblasts (Figure 2A) using WGA-594. We observed a ~20% increase in the mean fluorescence intensity of Pt4′s fibroblasts compared to WT.

We investigated whether the affected and unaffected fibroblasts could also be rapidly discriminated using flow cytometry and WGA-488 for this disease. This was successfully verified in Pt4′s fibroblasts (~20% increase in MFI/MFI_WTmean_ with respect to WT) and in the case of Pt5′s fibroblasts (~50% increase in MFI/MFI_WTmean_ compared to WT) (Figure 2B).

### 3.3. NPC

**Figure 3:**
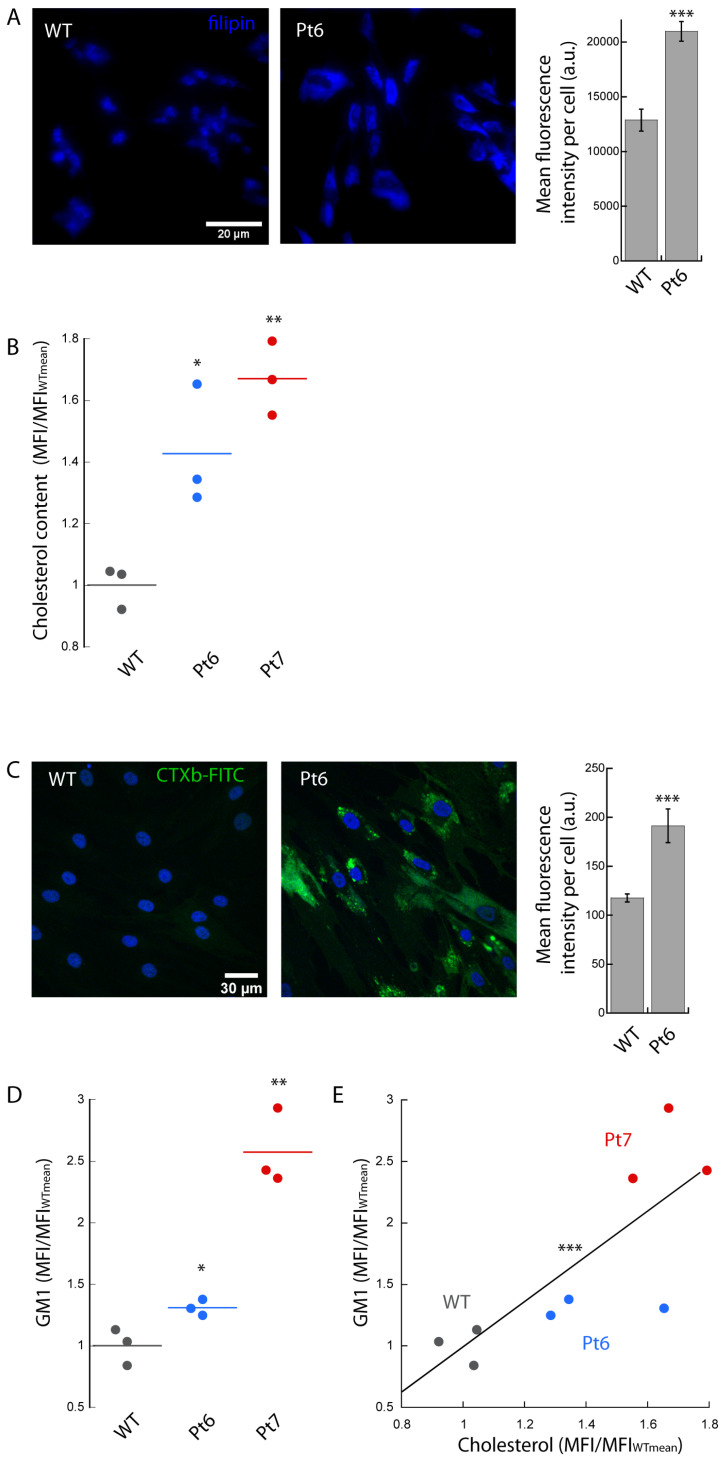
Type C Niemann–Pick disease. Thawed primary cultures of fibroblasts isolated from control and NPC patients (Pt6 and Pt7) were cultured, fixed, permeabilized, labeled with Filipin III (**A**,**B**) or CTXb-FITC (**C**,**D**), and imaged with wide field epifluorescence microscopy and CLSM, respectively (**A**,**C**), or analyzed with flow cytometry (**B**,**D**). Significantly higher values of Filipin III (**A**) and CTXb-FITC (**C**) fluorescence intensities were found analyzing Pt6 images compared to control, indicating a rise in cellular cholesterol and GM1 content, respectively. Scale bar 30 µm. >20 cells were analyzed for each condition. Error bar S.D. Flow cytometry MFI/MFI_WTmean_ values related to cholesterol (**B**) and GM1 (**D**) levels were found to increase in the fibroblast populations of Pt6 and Pt7 with respect to control. >5000 cells were analyzed for each MFI. Student’s *t*-test * *p* ≤ 0.05, ** *p* ≤ 0.01, *** *p* ≤ 0.001. (**E**) A significant linear correlation (R = 0.79, ** *p* ≤ 0.01) is found between the MFI/MFI_WTmean_ values relative to cholesterol and GM1 content.

Finally, we tested our approach on the fibroblasts isolated from patients with NPC disease. We proceeded, as in the two other disorders, testing if we could detect cholesterol accumulation, first with a wide field microscope, and then by flow cytometry. We used the standard Filipin III protocol to fluorescently label the cholesterol in the fixed and permeabilized fibroblasts from WT and patients. We observed a significant (~1.5-fold) increase in the total cholesterol levels with CLSM in Pt6′ fibroblasts compared to the control (Figure 3A).

We resuspended, fixed, and labeled the cells at flow cytometry, and we found a ~1.4-fold increase in the MFI/MFI_WTmean_ values for Pt6 with respect to WT (Figure 3B). In the case of Pt7, we found a ~1.7-fold higher MFI/MFI_WTmean_ values compared to control.

We also evaluated the levels of GM1 in NPC patients by using CTXb-FITC. The CLSM imaging showed a significant (~1.6-fold) increase in GM1 content in Pt6 compared to the control, very close to the relative accumulation observed for cholesterol (Figure 3C). In agreement with the cholesterol data, flow cytometry analysis revealed a corresponding increase in the MFI/MFI_WTmean_ values relative to GM1 in Pt6 (~1.3-fold) and Pt7 (~2.6-fold) compared to WT (Figure 3D).The significant linear correlation between the MFI/MFI_WTmean_ values obtained for cholesterol and GM1 (Figure 3E) indicates that the fibroblasts with higher cholesterol content are very likely to bear a proportional accumulation of GM1.

## 4. Discussion

A diagnosis of sialidosis, GM1 gangliosidosis, or NPC should be considered when specific clinical signs and enzyme assay and/or genetic testing confirm clinical suspicion [29]. However, juvenile–adult forms, as well as attenuated forms, are difficult to recognize clinically. Genetic tests can reveal variants of unknown significance (VUS), which can be difficult to interpret [6].

In this context, it is crucial to assess potential biomarkers that could aid diagnosis and help evaluate disease progression [6]. Filipin staining, which measures the accumulation of unesterified cholesterol, is the gold standard for diagnosing NPC. However, data analysis remains operator-dependent [2]. In addition to the less sensitive and specific method of thin-layer chromatography, a new tandem mass spectrometry method for urinary oligosaccharides screening, able to diagnose several lysosomal diseases, including sialidosis and GM1 gangliosidosis, has been proposed [30], however requiring specialized centers and personnel.

Biomarkers are also essential for monitoring and assessing outcomes in ongoing clinical trials [6]. Currently, FDA-approved treatments for patients with GM1 gangliosidosis and sialidosis [31,32] are limited to supportive care. The efficacy of substrate reduction therapy, pharmacological chaperones, enzyme replacement therapy, stem cell transplantation, and gene therapy for GM1 gangliosidosis is currently being evaluated [6,33]. There are several ongoing phase I/II clinical trials for GM1 gangliosidosis, evaluating the safety and efficacy of adeno-associated virus-mediated GLB1 delivery by intravenous injections [34,35]. Currently, several clinical trials are ongoing with different potential therapeutic agents (https://clinicaltrials.gov/ accessed on 5 August 2022), and Miglustat has been approved by the EU, Japan, and Canada, for treating progressive neurological complications in NPC patients. A robust new method using focused biomarkers would be useful for evaluating drug treatments in vitro [36]. In addition, many clinical changes occurring during clinical trials can be monitored by measuring differences in selected biomarkers [6].

Our investigations into the feasibility of using fluorescent in situ staining and flow cytometric procedures on fibroblasts to detect GM1 ganglioside storage in GM1 gangliosidosis, sialic acid in sialidosis, and cholesterol and GM1 in NPC yielded encouraging results. Specific and easy-to-perform tests for measuring sialic acid, GM1 ganglioside, and filipin, selectively accumulated in sialidosis, GM1 gangliosidosis, and NPC, are essential in routine diagnostic practice. Biochemical/functional studies to discriminate between pathogenic mutations and benign variants among the VUS should also be carried out. The methods reported here can be used for characterizing patient cell lines and clarifying the role of newly-discovered mutations.

We report data for both forms of GM1 gangliosidosis, severe/infantile and attenuated/ juvenile, and propose cut-off values that distinguish between the control vs. patient GM1 ganglioside values. Although sialidosis is rare, we established sialic acid levels in the fibroblast cell lines of two patients, Pt4 and Pt5, with the mild type-I form and the more severe type-II form (see Table 1). In contrast to the results obtained for GM1 ganglioside, the data clearly discriminate between the patients and controls in both forms of sialidosis. The NPC1-causative mutations present in the NPC-selected cell lines reported here were previously detected at a homozygous level in patients with the severe infantile form [27]. However, Pt7, affected by NPC, and showing major cholesterol storage, did not present a fatal neonatal onset (Table 1), suggesting that additional analyses, including patients with attenuated NPC forms, are required. We also established that GM1 ganglioside could be detected in NPC patient fibroblasts. Our data are supported by several reports showing that NPC1-deficient cells accumulate gangliosides and other glycosphingolipids, leading to neuropathological abnormalities resembling those observed in gangliosidoses [37]. Considering that fresh biological samples for these diseases are rarely available, our finding that frozen/thawed fibroblasts, obtained, for example, from biobanks, can retain accumulation levels of metabolites is important, as it indicates that this type of cell could be used in preliminary drug screening.

We recently developed a simple system to monitor GM1 storage in peripheral lymphocytes by flow cytometry [9]. Similarly, the methods reported here could potentially track sialic acid and filipin in the lymphocytes of sialidosis and NPC patients, respectively.

A health surveillance program in UK children revealed that LSDs are amongst the most common causes of neurodegeneration (45% of cases) [38]. In addition, it has been demonstrated that the misregulation of GM1 content is directly involved in Huntington’s and Parkinson’s diseases, in cancer stem cells, and in mice cancer models [39]. We hypothesize that our robust and quick method, which detects GM1 ganglioside in fibroblasts and peripheral blood, could be used in the clinical assessment and management of several common neurodegenerative diseases.

## Figures and Tables

**Table 1:** Clinical and molecular genetic findings of analyzed patients.

Pt	Ph, *Mutated Gene*	Nucleotide Changes	Amino Acid Changes	Clinical Data and Ref.	Molecular Genetic Ref.
1.	GM1, J, *GLB1*	c.152T > A/ c.602G > A	p.Ile51Asn/ p.Arg201His	- Skeletal involvement - Corneal opacity [18]	[18,23]
2.	GM1, J, *GLB1*	c.602G > A/ c.247dup1	p.Arg201His/ p.Tyr83LeufsX8	- Developmental regression - Dysarthria - Extrapyramidal symptoms - Cognitive impairment [19]	[19,23]
3.	GM1, I, *GLB1*	c.176G > A/ c.176G > A	p.Arg59His/ p.Arg59His	- Psychomotor impairment - Dysostosis multiplex - Hepatosplenomegaly - Cardiomyopathy [20]	[24]
4.	Sial I, *NEU1*	c.880C > T/ c.1004C > A	p.Arg294Cys/ p.Pro335Gln	- Myoclonus - Seizure - Cerebellar atrophy - Ataxia - Impaired vision [11]	[25]
5.	Sial II, *NEU1*	c.679G > A/ c.679G > A	p. Gly227Arg/ p. Gly227Arg	- Psychomotor delay - Hearing loss - Dysostosis multiplex - Seizures [21]	[26]
6.	NPC, *NPC1*	c.3613dup/ c.3613dup	p.Thr1205Asnfs*53fs/ p.Thr1205Asnfs*53fs	- Hypotonia - Jaundice - Splenomegaly - Neonatal onset (0–1 m.) - Failure to thrive - Hepatomegaly	[27]
7.	NPC, *NPC1*	c.2972_2973delAG/ c.2972_2973delAG	p.Q991Rfs*15/ p.Q991Rfs*15	- Regression of motor and psychic functions - Hypotonia - Splenomegaly - Late infancy onset (1–6 y)	[28]

Legend. Pt = patient; Ph = phenotype; GM1 = GM1 gangliosidodis; I = Infantile; J = Juvenile; Sial I = sialidosis type I; Sial II = sialidosis type II; NPC = Niemann–Pick disease, type C.

## Data Availability

Not applicable.
